# Single-Nucleotide Resolution Mapping of Hepatitis B Virus Promoters in Infected Human Livers and Hepatocellular Carcinoma

**DOI:** 10.1128/JVI.01625-16

**Published:** 2016-11-14

**Authors:** Kübra Altinel, Kosuke Hashimoto, Yu Wei, Christine Neuveut, Ishita Gupta, Ana Maria Suzuki, Alexandre Dos Santos, Pierrick Moreau, Tian Xia, Soichi Kojima, Sachi Kato, Yasuhiro Takikawa, Isao Hidaka, Masahito Shimizu, Tomokazu Matsuura, Akihito Tsubota, Hitoshi Ikeda, Sumiko Nagoshi, Harukazu Suzuki, Marie-Louise Michel, Didier Samuel, Marie Annick Buendia, Jamila Faivre, Piero Carninci

**Affiliations:** a RIKEN Center for Life Science Technologies, Division of Genomic Technologies, Yokohama, Kanagawa, Japan; b Laboratoire de Pathogenèse des Virus de l'Hépatite B, Institut Pasteur, Paris, France; c Hepacivirus et Immunité Innée, UMR CNRS 3569, Institut Pasteur, Paris, France; d INSERM, U1193, Paul-Brousse Hospital, Hepatobiliary Centre, Villejuif, France; e Université Paris Sud, Faculté de Médecine Le Kremlin Bicêtre, Villejuif, France; f RIKEN Center for Life Science Technologies, Division of Bio-function Dynamics Imaging, Wako, Saitama, Japan; g Department of Internal Medicine, Iwate Medical University, Iwate, Japan; h Department of Gastroenterology and Hepatology, Yamaguchi University Graduate School of Medicine, Yamaguchi, Japan; i Department of Gastroenterology/Internal Medicine, Gifu University Graduate School of Medicine, Gifu, Japan; j Department of Laboratory Medicine, Jikei University School of Medicine, Tokyo, Japan; k Research Center for Medical Science, Jikei University School of Medicine, Tokyo, Japan; l Department of Clinical Laboratory Medicine, The University of Tokyo, Tokyo, Japan; m Department of Gastroenterology and Hepatology Saitama Medical Center, Saitama Medical University, Saitama, Japan; n Assistance Publique-Hôpitaux de Paris, Pôle de Biologie Médicale, Paul-Brousse Hospital, Villejuif, France; University of Florida

## Abstract

Hepatitis B virus (HBV) is a major cause of liver diseases, including hepatocellular carcinoma (HCC), and more than 650,000 people die annually due to HBV-associated liver failure. Extensive studies of individual promoters have revealed that heterogeneous RNA 5′ ends contribute to the complexity of HBV transcriptome and proteome. Here, we provide a comprehensive map of HBV transcription start sites (TSSs) in human liver, HCC, and blood, as well as several experimental replication systems, at a single-nucleotide resolution. Using CAGE (cap analysis of gene expression) analysis of 16 HCC/nontumor liver pairs, we identify 17 robust TSSs, including a novel promoter for the X gene located in the middle of the gene body, which potentially produces a shorter X protein translated from the conserved second start codon, and two minor antisense transcripts that might represent viral noncoding RNAs. Interestingly, transcription profiles were similar in HCC and nontumor livers, although quantitative analysis revealed highly variable patterns of TSS usage among clinical samples, reflecting precise regulation of HBV transcription initiation at each promoter. Unlike the variety of TSSs found in liver and HCC, the vast majority of transcripts detected in HBV-positive blood samples are pregenomic RNA, most likely generated and released from liver. Our quantitative TSS mapping using the CAGE technology will allow better understanding of HBV transcriptional responses in further studies aimed at eradicating HBV in chronic carriers.

**IMPORTANCE** Despite the availability of a safe and effective vaccine, HBV infection remains a global health problem, and current antiviral protocols are not able to eliminate the virus in chronic carriers. Previous studies of the regulation of HBV transcription have described four major promoters and two enhancers, but little is known about their activity in human livers and HCC. We deeply sequenced the HBV RNA 5′ ends in clinical human samples and experimental models by using a new, sensitive and quantitative method termed cap analysis of gene expression (CAGE). Our data provide the first comprehensive map of global TSS distribution over the entire HBV genome in the human liver, validating already known promoters and identifying novel locations. Better knowledge of HBV transcriptional activity in the clinical setting has critical implications in the evaluation of therapeutic approaches that target HBV replication.

## INTRODUCTION

Hepatitis B virus (HBV) is a major etiological agent of acute and chronic liver diseases, including hepatocellular carcinoma (HCC), the second leading cause of cancer mortality worldwide (1, 2). About 240 million people are estimated to be chronically infected by HBV, and more than 650,000 people die annually due to HBV-associated liver failure (3). HBV is the prototype member of the hepadnavirus family, characterized by a compact DNA genome replicating with its own reverse transcriptase from an RNA intermediate (4). This virus is classified into eight major genotypes with distinct geographic distributions (5, 6). The HBV genome carries four open reading frames (ORFs), which encode seven different proteins, including three surface proteins, the core and e antigens, the polymerase, and the X transactivator. The expression of these genes is regulated by four promoters and two enhancers, which direct the production of six distinct mRNAs (two 3.5-kb transcripts for the core and e antigens, the polymerase, and for pregenomic RNA (pgRNA), one 2.4-kb transcript for the large surface protein (LHBs), two 2.1-kb transcripts for the middle and small surface proteins (MHBs and SHBs, respectively), and one 0.7-kb mRNA for the X transactivator protein (7–9). Heterogeneous 5′ ends and in-frame ATG codons play important roles in increasing the diversity of proteins made from a small genome. For example, two 3.5-kb mRNAs are transcribed from the core promoter with slightly different transcription start sites (TSSs); one containing the preC start codon is named precore mRNA, translated into the precore protein and giving rise to the e antigen, and the other missing this start codon is called pgRNA, which is translated into the core and polymerase proteins, in addition to serving as a template for viral DNA replication (8, 10). In addition, two 2.1-kb mRNAs are transcribed from the S promoter with 5′ heterogeneity; one TSS containing the preS2 start codon is for MHBs, and the other downstream the preS2 start codon is for the SHBs (11). The exact positions of TSSs for individual promoters have been studied using 5′ RACE (rapid amplification of cDNA ends) and an RNase protection assay; however, how frequently each start site is used in different hosts and conditions is not well understood. In addition, analysis of the numerous TSSs in the HBV genome requires high-throughput technologies for comprehensive mapping in a quantitative manner.

Cap analysis of gene expression (CAGE), a method for genome-wide identification of TSSs, is focused on the selective capture of the capped 5′ ends of RNAs (cap trapping). It is based on the principle that the cap site and the 3′ end of mRNA are the only sites carrying the diol structure that can be chemically labeled with a biotin group. By using streptavidin-coated magnetic beads, only the full-length first-strand cDNA/mRNA hybrids are selectively recovered after RNase I treatment. Sequencing short sequence reads (or tags) taken from the 5′ ends of full-length cDNAs allows TSSs to be mapped and their expression, measured by tag frequency, to be analyzed (12, 13). The CAGE technology has been extensively used to identify exact positions of TSSs in various different organisms from mammals to viruses (14–16). CAGE has also shown high reproducibility for expression measurements through a number of large scale projects, including FANTOM (17, 18) and ENCODE (19), discovering novel noncoding RNAs (ncRNAs) and transcribed enhancers (20). Because the HBV genome is entirely coding, with extensive overlap of the viral genes, quantification of individual transcripts by RNA-Seq is not feasible. Here, we used the CAGE technology for quantitative mapping of TSSs on the whole HBV genome at a single-nucleotide resolution. We analyzed the HBV transcriptome in chronically infected human livers and HCCs that we collected and sequenced in a previous study (21) and in whole blood from HBV-positive patients, as well as several experimental models of HBV replication. To our knowledge, our data provide the most comprehensive map of quantitative TSSs as a resource to study transcriptional activity of HBV in experimental setting and design new therapeutic approaches for inhibiting HBV replication in chronically infected patients.

## MATERIALS AND METHODS

### CAGE libraries for human liver tissues.

CAGE libraries for human liver tissues were prepared and sequenced as detailed in our previous study (21). Raw data are available through the NCBI dbGaP database (22) under accession number phs000885.v1.p1 (controlled access). Briefly, liver tissues, including tumor and nontumor samples, were collected from patients resected for HCC (Table 1; see also Table S1 [*c18* http://gerg.gsc.riken.jp/JVI2016/SupplementaryMaterials.pdf]). CAGE libraries were prepared according to a published protocol (12) and sequenced with single-end reads of 50 bp on an Illumina HiSeq 2000 platform. The ethics evaluation committees of the INSERM (IRB00003888, FWA00005831) and RIKEN (H24-4) approved the use of human liver samples. All patients provided written informed consent.

**TABLE 1:** Clinical samples with serological HBV markers^*a*^

Sample	Gender	Age (yr)	Predicted genotype		HBsAg status	HBcAg, HBeAg, and/or anti-HBe status	Antiviral treatment
			T	NT			
1	Male	47	A	G	Positive	Negative	Lamivudine and adefovir dipivoxil
2	Male	62	E	A	Positive	Anti-HBe positive	Tenofovir
3	Male	56			Negative	Anti-HBc positive	No treatment
4	Male	59	D	D	Negative	Anti-HBc positive	No treatment
5	Male	73	D	D	Positive	HBeAg positive	Adefovir dipivoxil
6	Female	56	D	A	Positive	NA	Lamivudine
7	Male	54	D	E	NA	NA	NA
8	Male	38	A	A	Positive	Anti-HBc, anti-HBe positive	Lamivudine
9	Male	49	D	D	Positive	Anti-HBc, anti-HBe positive	No treatment
10	Male	60	E	E	Positive	HBeAg positive	Adefovir dipivoxil
11	Male	47	E	E	Positive	NA	Lamivudine
12	Male	58	C	C	Positive	HBeAg positive	Lamivudine
13	Male	73	D	D	Positive	NA	NA
14	Male	72	A	A	Negative	Anti-HBc positive	No treatment
15	Male	55	A	A	Positive	HBcAg positive	Lamivudine, adefovir dipivoxil, and tenofovir
16	Male	66	C	C	Positive	Anti-HBc positive	Lamivudine and adefovir dipivoxil

a HBcAg, HBcAg, HBeAg, and anti-HBe were measured in serum. NA, data not available.

### CAGE libraries for human blood and HBV model systems.

The ethics review committee of Saitama Medical University approved the use of human blood samples. Blood samples were collected from male HBV (genotype C) positive patients who did not develop HCC. Total RNA was extracted from RNAlater (Ambion)-treated whole blood using the RiboPure-Blood kit (Ambion), followed by the RNeasy kit (Qiagen), for further purification. CAGE libraries were prepared in accordance with the latest version of the protocol, which does not require a PCR amplification step (13), and were sequenced with single-end reads of 50 bp on the Illumina HiSeq 2000 platform.

### Determination of CAGE TSSs.

We mapped the CAGE tags to the human genome (hg19/GRCh37 assembly) or to the murine genome (mm9/NCBI37 assembly) in the case of the murine liver transduced with AAV-HBV, using BWA v0.5.9 (23) with default parameters on the MOIRAI pipeline (24). The unmapped tags extracted from the mapping results were aligned to 16 representative HBV genomes downloaded from HBVdb (25). Because the HBV genome is circular, each genome was tandemly repeated for mapping all genome sequences. After alignment of tag sequences with 16 representative HBV genomes, the HBV genotype in each sample was defined as the genome to which the highest tag counts were mapped. To unify the genomic positions of HBV, an HBV genome sequence (accession number GQ358158.1 in GenBank) with genotype C and genome size: 3,215 bp was selected as a reference genome. The genomic positions for the other genomes were converted to those of the reference genome based on multiple alignments of HBV genomes. CAGE technology often adds an extra G base to the 5′ end in the reverse transcription process (26). To correct one base shift of the 5′ end, the first mismatched G was removed. We then clustered the tags to define distinct CAGE peaks using Paraclu with the following parameters: (i) a minimum of 100 total tags per cluster, (ii) a minimum density increase of 2, and (iii) a maximal cluster length of 100 bp (27). Paraclu was designed to identify CAGE TSS peaks and is commonly used in studies using CAGE such as ENCODE. The algorithm calculates densities of CAGE tags and finds maximal segments where every prefix and suffix of the segment has a given density. Raw tag counts for each peak were divided by a total tag count of the library, including human transcriptome, to calculate normalized expression values. The unit of the expression value is tpm, i.e., tags per mapped million tags. CAGE tags and peaks were visualized using IGV (28).

### Cells.

The HepAD38 cell line is derived from HepG2 cells and contains the HBV genome (subtype ayw) under tetracycline control (29). HepAD38 cells were maintained in Dulbecco modified Eagle medium/F-12 with 10% fetal calf serum (FCS), 3.5 × 10^–7^ M hydrocortisone hemisuccinate, and insulin at 5 μg/ml. Primary human hepatocytes (PHHs) were purchased from Corning (catalog number 454541, lot number 399) and maintained in PHH medium (Corning catalog number 355056, Corning hepatocyte culture media kit, 500 ml) as recommended by the manufacturer.

### Virus production and infection of primary human hepatocytes.

For virus production, HepAD38 cells were grown in Williams E medium with 5% FCS, 7 × 10^−5^ M hydrocortisone hemisuccinate, insulin at 5 μg/ml, and 2% dimethyl sulfoxide. HBV particles were concentrated from the clarified supernatant by overnight precipitation with 5% PEG 8000 and centrifugation at 4°C for 60 min at 5,000 rpm. Titers of enveloped DNA-containing viral particles were determined by immunoprecipitation with an anti-preS1 antibody (kindly provided by C. Sureau), followed by qPCR quantification of viral RC DNA with the following primers: RC5′ (5′-CACTCTATGGAAGGCGGGTA-3′) and RC3′ (5′-TGCTCCAGCTCCTACCTTGT-3′) (30). Around 20 to 25% of HBV DNA measured in the cell supernatant was recovered in the preS1 immunoprecipitate, correlating with the finding of 25% of enveloped virions and 75% of naked capsids by using native agarose gel electrophoresis, transfer onto nitrocellulose, and hybridization with radiolabeled HBV probe and anti-HBs antibody as previously described (31). For infection, only enveloped DNA-containing viral particles (vp) were taken into account to determine the multiplicity of infection (MOI). PHHs were infected as previously described with normalized amounts of virus at an MOI of 500 vp/cell (32).

### Animal experiments.

The AAV-HBV vector has been described previously (33). Six-week-old FVB/NCrl male mice obtained from The Jackson Laboratory received a single tail vein injection of 5 × 10^10^ viral genomes (vg) of AAV-HBV vector or control vector AAV-GFP. Mice were sacrificed at 20 weeks postinjection. Total RNA was extracted from mouse livers with TRI Reagent (Sigma-Aldrich). The experimental procedures were approved by the Institut Pasteur (CHSCT 10.289), in accordance with French government regulations. Mice were bred in a pathogen-free environment at the Institut Pasteur animal facility in accordance with welfare criteria outlined in the *Guide for the Care and Use of Laboratory Animals*.

### Northern blot analysis.

Portions (20 μg) of total RNA was denatured by formaldehyde, run on 1% agarose gels in 20 mM phosphate (pH 7.0) and 1% formaldehyde, and blotted onto Hybond N+ in 20× SSC (1× SSC is 0.15 M NaCl plus 0.015 M sodium citrate). A DNA fragment covering the HBV genome was used as probe. DNA labeling and hybridization were performed using a DIG High Prime DNA labeling and detection starter kit II (Roche). RNA sizes were estimated according to a molecular weight ladder (RNA molecular weight marker I, DIG labeled; Sigma-Aldrich).

### Accession number(s).

Supplementary materials are accessible at http://gerg.gsc.riken.jp/JVI2016/SupplementaryMaterials.pdf. CAGE data for human HCC samples were released in the NCBI database of Genotypes and Phenotypes (dbGaP; http://www.ncbi.nlm.nih.gov/gap/) under accession number phs000885.v1.p1. CAGE data for human blood samples were released in the NBDC human database (http://humandbs.biosciencedbc.jp/en/) under hum0050. CAGE data for HBV model systems were released in the Gene Expression Omnibus (GEO; http://www.ncbi.nlm.nih.gov/geo/) under accession number GSE84186.

## RESULTS AND DISCUSSION

### Variable expression of HBV transcripts in human livers and HCC.

**FIG 1:**
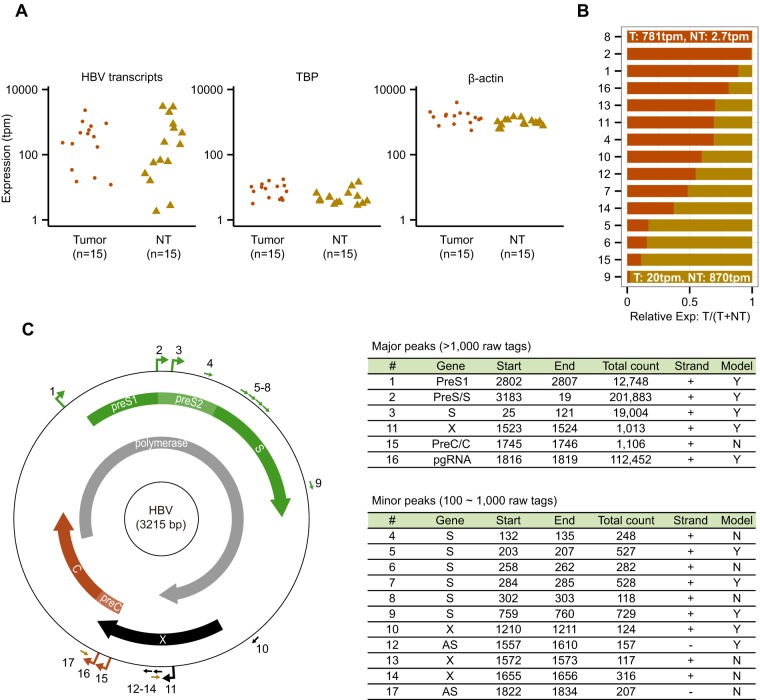
Quantification of HBV transcripts and comprehensive TSS map of HBV in chronically infected nontumor livers and HCCs. (A) Distribution of expression values for HBV transcripts and housekeeping genes in 15 tumor and 15 NT samples (sample 3 is excluded). The TATA box binding protein (*TBP*) and beta-actin (*ACTB*) genes are representatives of moderately and highly expressed genes, respectively. Expression data are derived from the same 15 HCC patients. (B) Relative HBV expression levels between tumor and matched nontumor samples. The relative value of 0.5 indicates that HBV expression levels are the same in T and NT. Samples are sorted by tumor ratios. (C) Distribution of detected CAGE peaks in the HBV genome. Large and small arrows on the outer circle indicate major CAGE peaks supported by >1,000 tags and minor peaks supported by 100∼1,000 tags. Arrows inside the circle represent open reading frames for four HBV genes. Genomic coordinates in the right panel correspond to the representative genome (GQ358158.1), where the EcoR1 site is the first position (+1). “Model” in the tables indicates whether the peak is present in HBV replication model systems (see Fig. 6 and see Table S6 in the supplemental material).

We have recently described CAGE analysis of human transcriptome in HCC and nontumor livers (21). Among analyzed cases, 16 pairs of tumor (T) and nontumor (NT) liver samples from HBV-positive HCC patients were selected for further studies of the HBV transcriptome (Table 1; see also Table S1 [*c18* http://gerg.gsc.riken.jp/JVI2016/SupplementaryMaterials.pdf]). Searching for CAGE tags that did not match human sequences, we identified a total of 376,770 tags that were mapped on the HBV genome using 16 representative HBV sequences curated by HBVdb (25) (see Table S2 in the supplemental material). Sequences from other viruses could not be found. HBV transcripts were detected in 30 of 32 samples with a 1 tag-per-million (tpm) threshold but not in HCV-associated samples used as controls. The expression levels of total HBV transcripts were highly variable in different samples from as low as TATA box binding protein (*TBP*) (median = 5.3 tpm) to as high as beta-actin (*ACTB*) (median = 1,128 tpm) (Fig. 1A). A high variability was observed also between T and NT samples from the same individual; for example, the expression value is 781 tpm in sample 8T and only 2.7 tpm in 8NT, the matched nontumor (Fig. 1B). It should be noted that most patients received antiviral treatment at the time of tumor resection (Table 1), which might account for low level of HBV transcription in a majority of samples, as previously reported (34). We then determined the closest genotype for each sample based on the counts of tags mapped on 16 HBV genomes across eight major genotypes (25). The determined genotypes with the highest tag counts were mostly A and D, which are known to be prevalent in Europe (Table 1). Despite a number of ambiguous sequence positions in the tags, we found evidence for different genotypes in four T/NT pairs, probably reflecting intergenotype dual infection of these patients as already reported in other studies (35).

**FIG 2:**
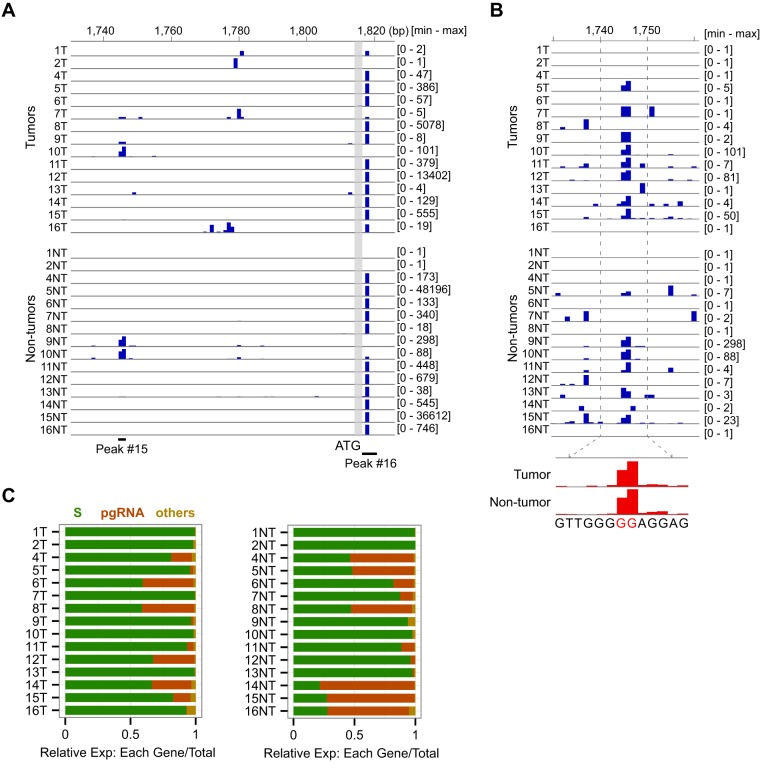
TSSs distribution in the basal core promoter (BCP) region in nontumor liver and HCC. (A) Major peaks for preC and pgRNA, showing a predominant peak for pgRNA (peak 16) at position 1818, and scattered minor peaks over the BCP region. The scale shown at the right represents the maximal tag counts in each sample. (B) Recurrent TSS for preC RNA (peak 15). The TSS shape from pooled samples is shown in the lower panel. (C) Relative expression levels of the preS/S transcripts, pgRNA, and others in T (left panel) and NT samples (right panel).

We identified 17 robust TSSs (CAGE peaks) supported by at least 100 raw tag counts in total and expressed at least in 10 samples, of which six peaks supported by more than 1,000 raw tags were referred to as major peaks (Fig. 1C). Several known TSSs were correctly identified as major peaks by this method, including preS1 (peak 1), preS2/S (peak 2), and pgRNA (peak 16). The TSS positions were either highly specific, especially for pgRNA at position 1818, 4 bp downstream of the preC start codon (7) (Fig. 2A), or scattered over several positions as exemplified by the basal core promoter (BCP) region between positions 1740 and 1785, where the preC RNA TSS has been mapped previously (8, 10). In this study, the only recurrent TSS position in the BCP region was localized at nucleotides 1745 and 1746 (Fig. 2B). In a large majority of T and NT samples, pgRNA levels were >10-fold higher than other transcripts in the BCP. As expected, preS/S transcripts and pgRNA were predominant RNAs in all samples (Fig. 2C), although the percentages varied greatly between individual cases. Among the minor peaks, two antisense transcripts that could represent ncRNAs were detected at low levels of expression (see Fig. 1C). One of them started in the preC region, close to the core promoter (peak 17), and the second, transcribed from the middle of the X gene (peak 12) and detected in 14 samples, was previously reported as a minor nonpolyadenylated transcript (36). Note that CAGE can capture both poly(A)^+^ and poly(A)^−^ transcripts by using random primers. These minor antisense transcripts might be involved in the regulation of the neighboring promoters.

### Heterogeneous promoter usage for the S and X genes.

**FIG 3:**
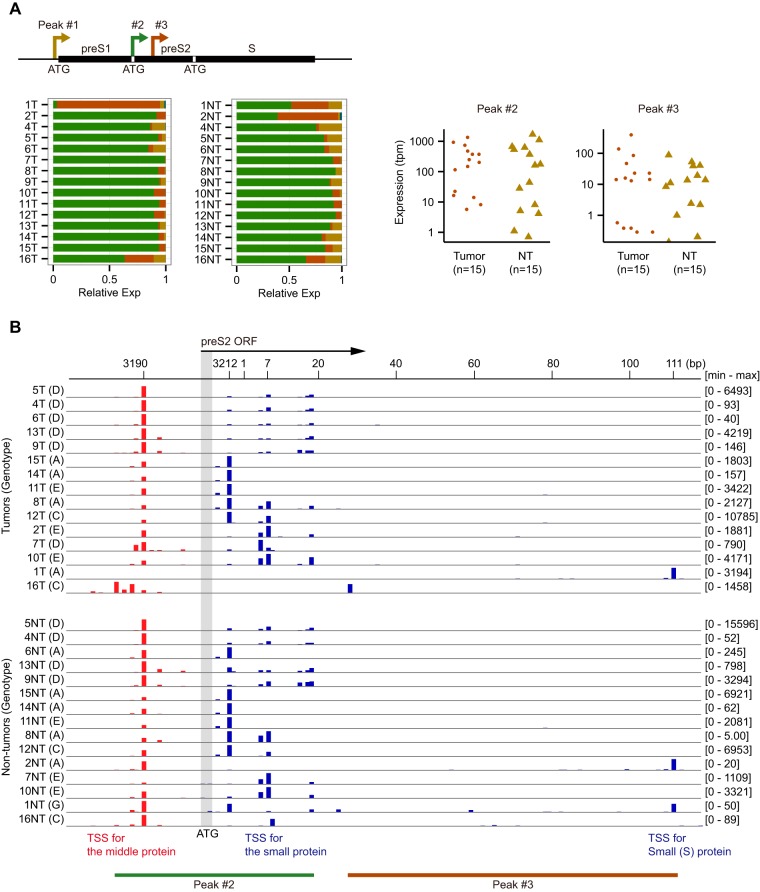
TSSs in the S region at a single-nucleotide resolution. (A) The major peaks for the S region. The peak 1 is located upstream of the preS1 ORF, whereas peaks 2 and 3 are located inside preS1 and preS2 ORFs. Relative expression values of three major peaks in T and NT samples are shown (yellow, 1; green, 2; red, 3; and blue, other minor peaks [found only in some samples such as 1T and 2NT]). Expression values of peaks 2 and 3 are shown in the log scale. (B) TSSs distribution inside the peaks 2 and 3 at a single-nucleotide resolution. The preS2 ATG is shown as a vertical gray bar. Red bars upstream of the preS2 ATG represent TSSs for the middle protein, whereas blue bars downstream of the ATG represent TSSs for the small protein.

It is known that two independent promoters are responsible for producing three forms of the surface proteins (large, middle, and small). The first one, called the preS1 promoter, characterized by a canonical TATA box, is located upstream of the first start codon. The other one, called the preS/S promoter, is located in the preS1 region, and it is devoid of a TATA box, which enables to generate two types of mRNAs, initiated either upstream or downstream of the preS2 start codon, and giving rise to the middle protein or the small protein using the third start codon (11). In addition to the preS1 promoter (peak 1) and the preS2/S promoter (peak 2), we identified an extra major promoter (peak 3) for the small protein (Fig. 3A). This potential novel promoter is characterized by an enriched TSS at position 111, immediately downstream of the preS2/S promoter, and it gives broad but weak signals (Fig. 3B). The expression level of this promoter is as high as the preS1 promoter, but much lower than the preS2/S promoter except for 1T and 2NT samples (Fig. 3A). Due to the predominant expression of the preS2/S promoter in a large majority of samples, the ratio of middle to small surface proteins depends on the TSS usage within this promoter. As reported in previous studies, we observed heterogeneous 5′ ends distributed upstream and downstream of the ATG but, more importantly, these TSSs are not equally used in different samples. Interestingly, our quantitative TSS mapping shows at least three distinct patterns of the TSS usage depending, at least in part, on the HBV genotype. The first group (genotype D) has the most frequent start site at nucleotide 3190 with weaker signals at nucleotides 3212, 5, 7, and 18. The second group (genotype A) has the strongest signal at nucleotide 3212 with a weaker signal at 3190, and the third group (mostly genotype E) has the strongest signal at nucleotides 5 or 7, with weaker signals at nucleotides 3190 and 3212 (Fig. 3B).

**FIG 4:**
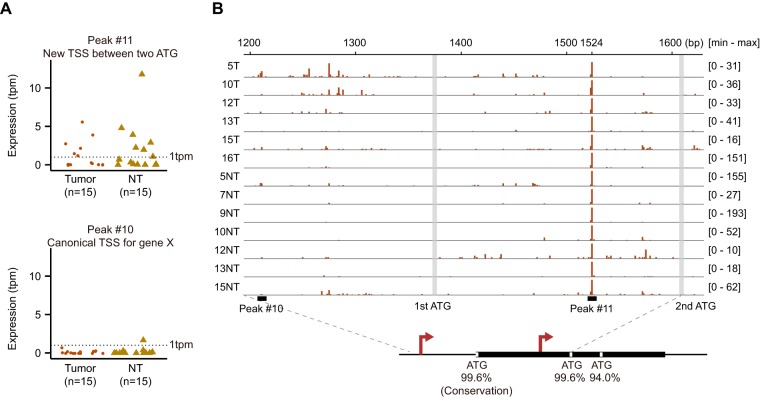
TSSs in the X gene region at a single-nucleotide resolution. (A) Expression values of the new TSS located inside the X gene (peak 11) and the canonical TSS upstream of the X gene (peak 10). (B) TSSs distribution between the first and second ATGs of the X gene for six tumors and seven nontumors in which the expression values are >1 tpm. The conservations of the three ATG among 6,949 nucleotide sequences from different HBV genotypes are 99.6% (6,919 of 6,949) 99.6% (6,919 of 6,949), and 94.0% (6,534 of 6,949).

Unlike the S gene, the X gene is widely believed to produce a single form of protein (17-kDa X protein or HBx) translated from a 0.7-kb mRNA. This protein is required for HBV replication *in vivo* and functions as a broad-range transactivator that stimulates the expression of viral and cellular genes (37–39). We identified a faint peak upstream of the first ATG of the X gene (peak 10, Fig. 4A), corresponding to a part of the canonical X promoter (9). Surprisingly, we identified a higher peak between the first and the second ATG (peak 11), which shows moderate expression levels in a subset of samples (Fig. 4A). The transcription starts preferentially at nucleotide 1524 in tumors and nontumor livers (Fig. 4B). Note that although the cap selectivity of the CAGE technology is very high (ca. 333- to 625-fold enrichment for capped RNAs [26]), it is still possible to have artificial peaks on cleavage hot-spots produced by massive amounts of site-specific cleavage events. Nevertheless, a recent study using covalently closed circular DNA (cccDNA) ChIP-Seq approach has shown two distinct peaks of active promoter marks (H3K4me3, H3K27ac, and H3K122ac) within the X gene body, especially prominent in HBV-infected primary human hepatocytes and HBV-positive liver tissues (40). The second histone modification peak located at the middle of the X gene might be associated with the new TSS detected here. Moreover, analysis of the conservation of X gene in-frame ATGs using 6,949 nucleotide sequences across eight genotypes showed that the second ATG is as well conserved as the first ATG (99.6% for both), whereas the third ATG is slightly less conserved (94.0%). It has been shown previously that the X gene is able to produce shorter peptides, which are translated from the second and the third in-frame start codons in cell lines, and can function as transactivators in a similar manner as full-length HBx (41, 42). Collectively, these data indicate that the potential novel transcript evidenced by CAGE might give rise to a shorter HBx protein retaining transactivator function to regulate viral and host genes.

### Comparison of TSS usage between HCCs and nontumor livers.

HBV expression levels were highly heterogeneous in HCC samples, with total counts ∼2-fold lower than those for nontumor liver samples (no significant difference, *P* = 0.7437, Wilcoxon rank-sum test). These data are in agreement with the notion that HBV transcription and replication are reduced in tumors compared to liver. As shown in Table S3 in the supplemental data (*c18* http://gerg.gsc.riken.jp/JVI2016/SupplementaryMaterials.pdf), predominant TSSs in HCCs were found for the preS2/S promoter (peak 2), followed by the core (peak 16), preS1 (peak 1) and S promoters (peak 11). Although the average activity of the preS2/S promoter was similar in T and NT livers, the average transcription from the preS1, core, and X promoters was ∼4-fold lower in HCCs. This might reflect the strict requirement of these promoters for liver-enriched transcription factors, in contrast to the preS2/S promoter regulation by ubiquitous factors (43). Presently, it is not possible to determine whether viral RNAs detected in HCC are produced from integrated HBV sequences or from episomal cccDNA. However, the TSS positions in HCCs were almost identical to those in NT livers, and we observed in most tumors a robust pgRNA TSS at position 1818, a region frequently disturbed by host-viral junctions upon HBV integration (44), suggesting that transcription might occur from the HBV cccDNA as well as integrated HBV sequences, which might contribute to HBV recurrence after liver transplantation (45).

### HBV transcriptome in blood.

**FIG 5:**
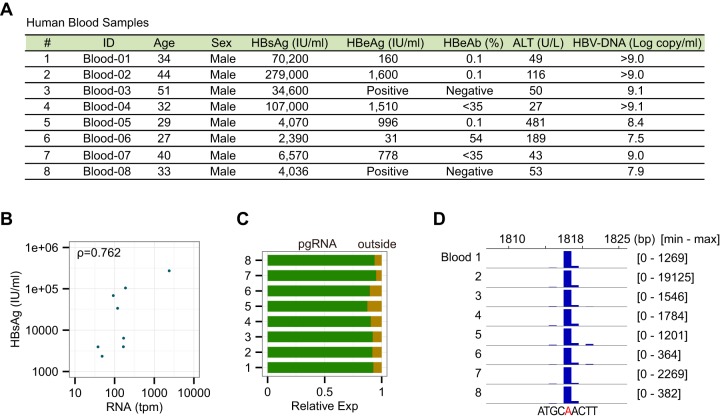
Identification and quantification of HBV transcripts in blood. (A) List of human blood samples analyzed by CAGE. Samples were collected from male genotype C-positive patients with various HBsAg levels. (B) Association between RNA and HBsAg levels in blood. Each dot represents one blood sample. Both *x* and *y* axes are shown in log-scale. (C) Relative expression of detected RNAs in blood. About 87 to 95% of the tags are mapped within the pgRNA peak. “Outside” indicates tags mapped outside all 17 HBV peaks detected in the liver transcriptome. (D) Transcription start sites of the pgRNA in blood. The scale of the *y* axis is fit to the raw tag counts of each sample.

The HBV genome is generally considered to be transcribed only in liver, but viral RNAs are frequently found in the sera of HBV-infected patients (46). To better characterize these HBV RNA species, we sequenced new CAGE libraries for eight whole-blood samples derived from HBV genotype C-positive patients, independent of the first patient group (Fig. 5A). CAGE tags aligned to the HBV genome (GQ358158.1, a representative genotype C) were detected from all samples, ranging from 40 to 2,500 tpm. The level of transcripts is significantly correlated with the level of HBsAg in blood (Spearman's correlation coefficient = 0.762 and *P* = 0.037) (Fig. 5B). Comparison of TSS positions with the 17 peaks identified in liver tissues showed a high prevalence of TSS at position 1818, whereas few tags were mapped to the S promoters (Fig. 5C and D). This major start site is consistent with the pgRNA TSS found in tumor and liver samples (see Fig. 2A), as well as in previous studies (7). Thus, the predominance of pgRNA in blood samples suggests that immature capsids, in which HBV DNA replication has not been completed, might be released from liver into blood, as already observed in several reports (46–49). We have to consider another possibility, where CAGE captures the capped 5′ end of the pgRNA that survives as a short RNA fragment attached to the “+polarity” DNA strand in HBV virions, although a majority of DNAs hybridized with short 5′ RNA is likely to be removed in the process of RNA purification and CAGE library preparation.

### HBV transcriptome in experimental model systems.

**FIG 6:**
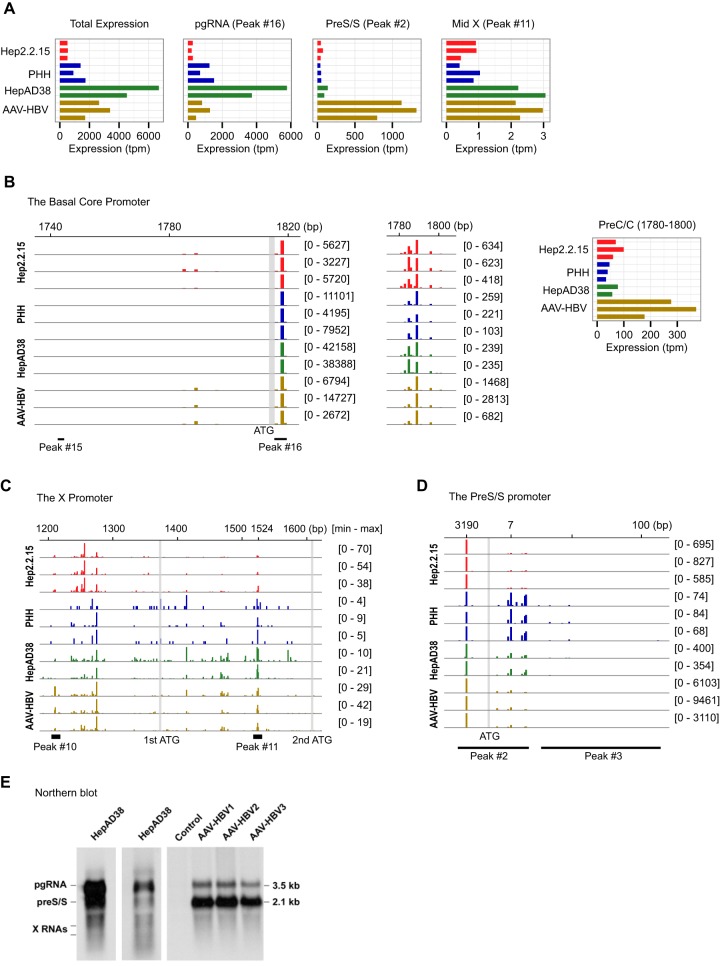
Identification and quantification of HBV transcripts in model systems. (A) Expression values of total HBV, pgRNA (peak 16), preS/S (peak 2), and the new TSS inside the X gene (peak 11). (B) TSS distribution in the basal core promoter region. Detailed CAGE signals and expression values between bp 1780 and 1800 are shown in the right panel. (C) TSSs distribution in the X promoter region upstream and downstream of the first start codon. (D) TSSs distribution in the preS/S promoter region. (E) Northern blot analysis of HBV RNA. The total RNA (20 μg) from HepAD38 cells and from mouse livers transduced with either AAV-HBV vector (AAV-HBV) or control AAV-GFP vector (control) was analyzed by Northern blotting. For HepAD38 cells, the results for a shorter exposure are shown in the second lane. The sizes of major transcripts are indicated on the right.

To determine whether HBV activities might be different in clinical and experimental conditions, we prepared and sequenced CAGE libraries for four experimental HBV model systems: (i) the HepG2.2.15 cell line, (ii) primary human hepatocytes (PHHs) infected with HBV, (iii) the HepAD38 cell line, and (iv) mouse liver transduced with AAV-HBV (see Table S4 in the supplemental material [*c18*; http://gerg.gsc.riken.jp/JVI2016/SupplementaryMaterials.pdf]). The PHH is a model in which HBV is transcribed from cccDNAs, whereas in the other three models, genes are mainly transcribed from integrated HBV genomes, although in HepAD38 cells, transcription might also occur from cccDNA that is made by nuclear reimport of encapsidated RCDNA (50). We identified HBV transcripts from all samples, ranging from 500 to 7,000 tpm (Fig. 6A), which is comparable with mean expressions of 523 tpm for tumors and 704 tpm for nontumors. We calculated expression values for the predefined 17 TSSs (see Table S5 in the supplemental material). Although pgRNA and preS/S RNAs are the major transcripts in all models, major differences between their relative levels are seen among the models. For example, the expression of preS/S is as high as pgRNA in the mouse AAV-HBV model, whereas the expression of pgRNA is predominant in the other models (Fig. 6A). We then independently performed peak calling for the model systems using Paraclu with the same parameters used for the clinical samples. We identified 15 peaks, 10 of which overlap the clinical peaks (5 of 6 major peaks and 5 of 11 minor peaks), indicating that a majority of TSS peaks are shared in clinical and experimental conditions (Fig. 1C and see Table S6 in the supplemental material). On the other hand, a major difference was found in the basal core promoter region, in which the major peak 15 was not detected in the model systems, instead clear signals were detected between bp 1780 and 1800 (Fig. 6B), corresponding to previous report of the preC RNA start site (10). The relative expression of the region from bp 1780 to 1800 among model systems is similar to the preS/S (peak 2), where the AAV-HBV model is the highest. The X promoter also shows a different pattern from the clinical condition. In addition to peaks 10 and 11, another broad peak was detected at around bp 1250, upstream of the 1st ATG, which can contribute to the full-length X protein (Fig. 6C). This suggests that HBV might use different promoters for two sizes of X proteins in different conditions, although functional analysis such as reporter assays is essential for further understanding of the promoter usage. The preS/S promoter shows consistent pattern with the clinical peaks for genotype D with some variable signals at bp 7 (Fig. 6D). Finally, we analyzed HBV RNA by Northern blotting in two experimental models, including HepAD38 cells and mouse liver transduced with AAV-HBV to visualize major HBV transcripts. The data showing important differences in the relative levels of major transcripts (pgRNA and preS2/S RNA) between the two systems are in complete agreement with the CAGE data (Fig. 6E).

### Conclusion.

In this study, we used CAGE analysis to investigate the HBV transcriptome in nontumor liver, HCC, and blood from HBV-positive patients, as well as in four experimental HBV replication systems. This approach provided extensive and accurate positioning of all HBV TSSs, as well as quantitative evaluation of relative expression levels in the clinical setting. We also provide evidence for HBV transcriptional activity in tumor cells, although transcription template (episomal or integrated HBV sequences) cannot be ascertained by current technologies. A comprehensive and quantitative map of HBV transcripts in human tissues has not been described so far using conventional technologies, due to the compact structure of the HBV genome and the overlap of different open reading frames. Here, transcription from the four well-studied promoters (core, preS1, S, and X promoters) was correctly detected in the regions described in previous studies, with detailed information on relative TSS usage in the S and core promoter regions. In addition, 11 novel TSSs were discovered. One novel major TSS is located in the X gene body between the first and second start codons. Because of the high reproducibility of this transcription peak, its higher expression level compared to the canonical X transcript in nearly all clinical samples, and the strong conservation of the second ATG, we propose this transcript as a candidate mRNA that encodes a shorter form of the X protein. It has been reported that this short X protein might be endowed with transactivator functions similar to full-length HBx (42). We also detected minor, recurrent antisense TSSs in the core promoter and the X gene, which might represent ncRNAs implicated in the regulation of HBV transcription and could be used for therapeutic approaches based on selective inhibition of HBV transcription and replication. Current therapeutic regimens, the use of including nucleos(t)ide analogs such as lamivudine, potently inhibit viral replication but are not capable of eliminating the virus or controlling infection on the long-term after drug withdrawal because of frequent persistence of the HBV cccDNA within hepatocytes. Therefore, a potential therapeutic strategy to eradicate HBV could be to silence and eliminate cccDNA from infected cells. In this context, our study offers new tools for the characterization of HBV transcriptional responses in experimental setting.

## References

[1] El-Serag HB 2012 Epidemiology of viral hepatitis and hepatocellular carcinoma. Gastroenterology 142:1264–1273. doi:10.1053/j.gastro.2011.12.061. 22537432 PMC3338949

[2] Neuveut C, Wei Y, Buendia MA 2010 Mechanisms of HBV-related hepatocarcinogenesis. J Hepatol 52:594–604. doi:10.1016/j.jhep.2009.10.033. 20185200

[3] World Health Organization. 2015 Guidelines for the prevention, care and treatment of persons with chronic hepatitis B Infection. World Health Organization, Geneva, Switzerland. 26225396

[4] Beck J, Nassal M 2007 Hepatitis B virus replication. World J Gastroenterol 13:48–64. doi:10.3748/wjg.v13.i1.48. 17206754 PMC4065876

[5] Schaefer S 2007 Hepatitis B virus taxonomy and hepatitis B virus genotypes. World J Gastroenterol 13:14–21. doi:10.3748/wjg.v13.i1.14. 17206751 PMC4065870

[6] Kramvis A 2014 Genotypes and genetic variability of hepatitis B virus. Intervirology 57:141–150. doi:10.1159/000360947. 25034481

[7] Will H, Reiser W, Weimer T, Pfaff E, Buscher M, Sprengel R, Cattaneo R, Schaller H 1987 Replication strategy of human hepatitis B virus. J Virol 61:904–911. 3806799 10.1128/jvi.61.3.904-911.1987 PMC254036

[8] Quarleri J 2014 Core promoter: a critical region where the hepatitis B virus makes decisions. World J Gastroenterol 20:425–435. doi:10.3748/wjg.v20.i2.425. 24574711 PMC3923017

[9] Yaginuma K, Nakamura I, Takada S, Koike K 1993 A transcription initiation site for the hepatitis B virus X gene is directed by the promoter-binding protein. J Virol 67:2559–2565. 8474161 10.1128/jvi.67.5.2559-2565.1993 PMC237576

[10] Chen IH, Huang CJ, Ting LP 1995 Overlapping initiator and TATA box functions in the basal core promoter of hepatitis B virus. J Virol 69:3647–3657. 7745713 10.1128/jvi.69.6.3647-3657.1995 PMC189080

[11] Siddiqui A, Jameel S, Mapoles J 1986 Transcriptional control elements of hepatitis B surface antigen gene. Proc Natl Acad Sci U S A 83:566–570. doi:10.1073/pnas.83.3.566. 3456153 PMC322904

[12] Takahashi H, Lassmann T, Murata M, Carninci P 2012 5′ end-centered expression profiling using cap-analysis gene expression and next-generation sequencing. Nat Protoc 7:542–561. doi:10.1038/nprot.2012.005. 22362160 PMC4094379

[13] Murata M, Nishiyori-Sueki H, Kojima-Ishiyama M, Carninci P, Hayashizaki Y, Itoh M 2014 Detecting expressed genes using CAGE. Methods Mol Biol 1164:67–85. doi:10.1007/978-1-4939-0805-9_7. 24927836

[14] Fort A, Hashimoto K, Yamada D, Salimullah M, Keya CA, Saxena A, Bonetti A, Voineagu I, Bertin N, Kratz A, Noro Y, Wong CH, de Hoon M, Andersson R, Sandelin A, Suzuki H, Wei CL, Koseki H, Consortium F, Hasegawa Y, Forrest AR, Carninci P 2014 Deep transcriptome profiling of mammalian stem cells supports a regulatory role for retrotransposons in pluripotency maintenance. Nat Genet 46:558–566. doi:10.1038/ng.2965. 24777452

[15] Haberle V, Li N, Hadzhiev Y, Plessy C, Previti C, Nepal C, Gehrig J, Dong X, Akalin A, Suzuki AM, van IWF, Armant O, Ferg M, Strahle U, Carninci P, Muller F, Lenhard B 2014 Two independent transcription initiation codes overlap on vertebrate core promoters. Nature 507:381–385. doi:10.1038/nature12974. 24531765 PMC4820030

[16] Taguchi A, Nagasaka K, Kawana K, Hashimoto K, Kusumoto-Matsuo R, Plessy C, Thomas M, Nakamura H, Bonetti A, Oda K, Kukimoto I, Carninci P, Banks L, Osuga Y, Fujii T 2015 Characterization of novel transcripts of human papillomavirus type 16 using cap analysis gene expression technology. J Virol 89:2448–2452. doi:10.1128/JVI.03433-14. 25505068 PMC4338893

[17] Carninci P, Kasukawa T, Katayama S, Gough J, Frith MC, Maeda N, Oyama R, Ravasi T, Lenhard B, Wells C, Kodzius R, Shimokawa K, Bajic VB, Brenner SE, Batalov S, Forrest AR, Zavolan M, Davis MJ, Wilming LG, Aidinis V, Allen JE, Ambesi-Impiombato A, Apweiler R, Aturaliya RN, Bailey TL, Bansal M, Baxter L, Beisel KW, Bersano T, Bono H, Chalk AM, Chiu KP, Choudhary V, Christoffels A, Clutterbuck DR, Crowe ML, 2005 The transcriptional landscape of the mammalian genome. Science 309:1559–1563. doi:10.1126/science.1112014. 16141072

[18] FANTOM Consortium, RIKEN PMI, RIKEN CLST, Forrest AR, Kawaji H, Rehli M, Baillie JK, de Hoon MJ, Haberle V, Lassmann T, Kulakovskiy IV, Lizio M, Itoh M, Andersson R, Mungall CJ, Meehan TF, Schmeier S, Bertin N, Jorgensen M, Dimont E, Arner E, Schmidl C, Schaefer U, Medvedeva YA, Plessy C, Vitezic M, Severin J, Semple C, Ishizu Y, Young RS, Francescatto M, Alam I, Albanese D, Altschuler GM, Arakawa T, Archer JA, Arner P, Babina M, Rennie S, Balwierz PJ, Beckhouse AG, Pradhan-Bhatt S, Blake JA, 2014 A promoter-level mammalian expression atlas. Nature 507:462–470. doi:10.1038/nature13182. 24670764 PMC4529748

[19] Encode Project Consortium. 2012 An integrated encyclopedia of DNA elements in the human genome. Nature 489:57–74. doi:10.1038/nature11247. 22955616 PMC3439153

[20] Andersson R, Gebhard C, Miguel-Escalada I, Hoof I, Bornholdt J, Boyd M, Chen Y, Zhao X, Schmidl C, Suzuki T, Ntini E, Arner E, Valen E, Li K, Schwarzfischer L, Glatz D, Raithel J, Lilje B, Rapin N, Bagger FO, Jorgensen M, Andersen PR, Bertin N, Rackham O, Burroughs AM, Baillie JK, Ishizu Y, Shimizu Y, Furuhata E, Maeda S, Negishi Y, Mungall CJ, Meehan TF, Lassmann T, Itoh M, Kawaji H, Kondo N, Kawai J, Lennartsson A, Daub CO, Heutink P, Hume DA, Jensen TH, Suzuki H, Hayashizaki Y, Muller F, Consortium F, Forrest AR, Carninci P, Rehli M, Sandelin A 2014 An atlas of active enhancers across human cell types and tissues. Nature 507:455–461. doi:10.1038/nature12787. 24670763 PMC5215096

[21] Hashimoto K, Suzuki AM, Dos Santos A, Desterke C, Collino A, Ghisletti S, Braun E, Bonetti A, Fort A, Qin XY, Radaelli E, Kaczkowski B, Forrest AR, Kojima S, Samuel D, Natoli G, Buendia MA, Faivre J, Carninci P 2015 CAGE profiling of ncRNAs in hepatocellular carcinoma reveals widespread activation of retroviral LTR promoters in virus-induced tumors. Genome Res 25:1812–1824. doi:10.1101/gr.191031.115. 26510915 PMC4665003

[22] Tryka KA, Hao L, Sturcke A, Jin Y, Wang ZY, Ziyabari L, Lee M, Popova N, Sharopova N, Kimura M, Feolo M 2014 NCBI's database of genotypes and phenotypes: dbGaP. Nucleic Acids Res 42:D975–D979. doi:10.1093/nar/gkt1211. 24297256 PMC3965052

[23] Li H, Durbin R 2009 Fast and accurate short read alignment with Burrows-Wheeler transform. Bioinformatics 25:1754–1760. doi:10.1093/bioinformatics/btp324. 19451168 PMC2705234

[24] Hasegawa A, Daub C, Carninci P, Hayashizaki Y, Lassmann T 2014 MOIRAI: a compact workflow system for CAGE analysis. BMC Bioinformatics 15:144. doi:10.1186/1471-2105-15-144. 24884663 PMC4033680

[25] Hayer J, Jadeau F, Deleage G, Kay A, Zoulim F, Combet C 2013 HBVdb: a knowledge database for hepatitis B virus. Nucleic Acids Res 41:D566–D570. doi:10.1093/nar/gks1022. 23125365 PMC3531116

[26] Carninci P, Sandelin A, Lenhard B, Katayama S, Shimokawa K, Ponjavic J, Semple CA, Taylor MS, Engstrom PG, Frith MC, Forrest AR, Alkema WB, Tan SL, Plessy C, Kodzius R, Ravasi T, Kasukawa T, Fukuda S, Kanamori-Katayama M, Kitazume Y, Kawaji H, Kai C, Nakamura M, Konno H, Nakano K, Mottagui-Tabar S, Arner P, Chesi A, Gustincich S, Persichetti F, Suzuki H, Grimmond SM, Wells CA, Orlando V, Wahlestedt C, Liu ET, Harbers M, Kawai J, Bajic VB, Hume DA, Hayashizaki Y 2006 Genome-wide analysis of mammalian promoter architecture and evolution. Nat Genet 38:626–635. doi:10.1038/ng1789. 16645617

[27] Frith MC, Valen E, Krogh A, Hayashizaki Y, Carninci P, Sandelin A 2008 A code for transcription initiation in mammalian genomes. Genome Res 18:1–12. 18032727 10.1101/gr.6831208 PMC2134772

[28] Thorvaldsdottir H, Robinson JT, Mesirov JP 2013 Integrative Genomics Viewer (IGV): high-performance genomics data visualization and exploration. Brief Bioinform 14:178–192. doi:10.1093/bib/bbs017. 22517427 PMC3603213

[29] Ladner SK, Otto MJ, Barker CS, Zaifert K, Wang GH, Guo JT, Seeger C, King RW 1997 Inducible expression of human hepatitis B virus (HBV) in stably transfected hepatoblastoma cells: a novel system for screening potential inhibitors of HBV replication. Antimicrob Agents Chemother 41:1715–1720. 9257747 10.1128/aac.41.8.1715 PMC163991

[30] Cougot D, Allemand E, Riviere L, Benhenda S, Duroure K, Levillayer F, Muchardt C, Buendia MA, Neuveut C 2012 Inhibition of PP1 phosphatase activity by HBx: a mechanism for the activation of hepatitis B virus transcription. Sci Signal 5:ra1. 22215732 10.1126/scisignal.2001906

[31] Guo H, Jiang D, Zhou T, Cuconati A, Block TM, Guo JT 2007 Characterization of the intracellular deproteinized relaxed circular DNA of hepatitis B virus: an intermediate of covalently closed circular DNA formation. J Virol 81:12472–12484. doi:10.1128/JVI.01123-07. 17804499 PMC2169032

[32] Lucifora J, Arzberger S, Durantel D, Belloni L, Strubin M, Levrero M, Zoulim F, Hantz O, Protzer U 2011 Hepatitis B virus X protein is essential to initiate and maintain virus replication after infection. J Hepatol 55:996–1003. doi:10.1016/j.jhep.2011.02.015. 21376091

[33] Dion S, Bourgine M, Godon O, Levillayer F, Michel ML 2013 Adeno-associated virus-mediated gene transfer leads to persistent hepatitis B virus replication in mice expressing HLA-A2 and HLA-DR1 molecules. J Virol 87:5554–5563. doi:10.1128/JVI.03134-12. 23468504 PMC3648192

[34] Werle-Lapostolle B, Bowden S, Locarnini S, Wursthorn K, Petersen J, Lau G, Trepo C, Marcellin P, Goodman Z, Delaney WE, Xiong S, Brosgart CL, Chen SS, Gibbs CS, Zoulim F 2004 Persistence of cccDNA during the natural history of chronic hepatitis B and decline during adefovir dipivoxil therapy. Gastroenterology 126:1750–1758. doi:10.1053/j.gastro.2004.03.018. 15188170

[35] Amaddeo G, Cao Q, Ladeiro Y, Imbeaud S, Nault JC, Jaoui D, Gaston Mathe Y, Laurent C, Laurent A, Bioulac-Sage P, Calderaro J, Zucman-Rossi J 2015 Integration of tumour and viral genomic characterizations in HBV-related hepatocellular carcinomas. Gut 64:820–829. doi:10.1136/gutjnl-2013-306228. 25021421 PMC4392232

[36] Moriyama K, Hayashida K, Shimada M, Nakano S, Nakashima Y, Fukumaki Y 2003 Antisense RNAs transcribed from the upstream region of the precore/core promoter of hepatitis B virus. J Gen Virol 84:1907–1913. doi:10.1099/vir.0.19170-0. 12810886

[37] Murakami S 1999 Hepatitis B virus X protein: structure, function and biology. Intervirology 42:81–99. doi:10.1159/000024969. 10516464

[38] Nomura T, Lin Y, Dorjsuren D, Ohno S, Yamashita T, Murakami S 1999 Human hepatitis B virus X protein is detectable in nuclei of transfected cells, and is active for transactivation. Biochim Biophys Acta 1453:330–340. doi:10.1016/S0925-4439(99)00004-6. 10101251

[39] Williams JS, Andrisani OM 1995 The hepatitis B virus X protein targets the basic region-leucine zipper domain of CREB. Proc Natl Acad Sci U S A 92:3819–3823. doi:10.1073/pnas.92.9.3819. 7731990 PMC42053

[40] Tropberger P, Mercier A, Robinson M, Zhong W, Ganem DE, Holdorf M 2015 Mapping of histone modifications in episomal HBV cccDNA uncovers an unusual chromatin organization amenable to epigenetic manipulation. Proc Natl Acad Sci U S A 112:E5715–E5724. doi:10.1073/pnas.1518090112. 26438841 PMC4620859

[41] Zheng YW, Riegler J, Wu J, Yen TS 1994 Novel short transcripts of hepatitis B virus X gene derived from intragenic promoter. J Biol Chem 269:22593–22598. 8077209

[42] Kwee L, Lucito R, Aufiero B, Schneider RJ 1992 Alternate translation initiation on hepatitis B virus X mRNA produces multiple polypeptides that differentially transactivate class II and III promoters. J Virol 66:4382–4389. 1318408 10.1128/jvi.66.7.4382-4389.1992 PMC241245

[43] Seeger C, Mason WS 2000 Hepatitis B virus biology. Microbiol Mol Biol Rev 64:51–68. doi:10.1128/MMBR.64.1.51-68.2000. 10704474 PMC98986

[44] Sung WK, Zheng H, Li S, Chen R, Liu X, Li Y, Lee NP, Lee WH, Ariyaratne PN, Tennakoon C, Mulawadi FH, Wong KF, Liu AM, Poon RT, Fan ST, Chan KL, Gong Z, Hu Y, Lin Z, Wang G, Zhang Q, Barber TD, Chou WC, Aggarwal A, Hao K, Zhou W, Zhang C, Hardwick J, Buser C, Xu J, Kan Z, Dai H, Mao M, Reinhard C, Wang J, Luk JM 2012 Genome-wide survey of recurrent HBV integration in hepatocellular carcinoma. Nat Genet 44:765–769. doi:10.1038/ng.2295. 22634754

[45] Faria LC, Gigou M, Roque-Afonso AM, Sebagh M, Roche B, Fallot G, Ferrari TC, Guettier C, Dussaix E, Castaing D, Brechot C, Samuel D 2008 Hepatocellular carcinoma is associated with an increased risk of hepatitis B virus recurrence after liver transplantation. Gastroenterology 134:1890–1899. doi:10.1053/j.gastro.2008.02.064. 18424269

[46] van Bommel F, Bartens A, Mysickova A, Hofmann J, Kruger DH, Berg T, Edelmann A 2015 Serum hepatitis B virus RNA levels as an early predictor of hepatitis B envelope antigen seroconversion during treatment with polymerase inhibitors. Hepatology 61:66–76. doi:10.1002/hep.27381. 25132147

[47] Miller RH, Tran CT, Robinson WS 1984 Hepatitis B virus particles of plasma and liver contain viral DNA-RNA hybrid molecules. Virology 139:53–63. doi:10.1016/0042-6822(84)90329-5. 6495659

[48] Hatakeyama T, Noguchi C, Hiraga N, Mori N, Tsuge M, Imamura M, Takahashi S, Kawakami Y, Fujimoto Y, Ochi H, Abe H, Maekawa T, Kawakami H, Yatsuji H, Aisaka Y, Kohno H, Aimitsu S, Chayama K 2007 Serum HBV RNA is a predictor of early emergence of the YMDD mutant in patients treated with lamivudine. Hepatology 45:1179–1186. doi:10.1002/hep.21581. 17465002

[49] Wang J, Shen T, Huang X, Kumar GR, Chen X, Zeng Z, Zhang R, Chen R, Li T, Zhang T, Yuan Q, Li PC, Huang Q, Colonno R, Jia J, Hou J, McCrae MA, Gao Z, Ren H, Xia N, Zhuang H, Lu F 2016 Serum hepatitis B virus RNA is encapsidated pregenome RNA that may be associated with persistence of viral infection and rebound. J Hepatol 65:700–710. doi:10.1016/j.jhep.2016.05.029. 27245431

[50] Zhou T, Guo H, Guo JT, Cuconati A, Mehta A, Block TM 2006 Hepatitis B virus e antigen production is dependent upon covalently closed circular (ccc) DNA in HepAD38 cell cultures and may serve as a cccDNA surrogate in antiviral screening assays. Antiviral Res 72:116–124. doi:10.1016/j.antiviral.2006.05.006. 16780964

